# Reducing the maximal calcium conductance in models of the pyloric network after decentralization prevents recovery

**DOI:** 10.1186/1471-2202-13-S1-P67

**Published:** 2012-07-16

**Authors:** Claire Tang, Amber E Hudson, Astrid A Prinz

**Affiliations:** 1 Department of Biology, Emory University, Atlanta, GA 30322, USA; 2 Department of Biomedical Engineering, Georgia Institute of Technology, Atlanta, GA, 30332, USA

## 

The pyloric network is a small neural network found in the stomatogastric ganglion of decapod crustaceans that drives the contraction of a subset of their stomach muscles. It consists of different groups of neurons, with well-characterized intrinsic properties, that fire in a triphasic pattern because of the connectivity of the network [1]. The stomatogastric ganglion’s rhythmic activity is normally dependent on neuromodulatory input to the system. When that input is removed in a process called decentralization, such as by physically cutting the nerve that provides it, the neurons of the pyloric network stop firing. Over the course of the next several hours, the network begins firing in bouts before recovering to a rhythm similar to its original pattern of activity. For this recovery to occur, the network must have a set target level of activity, a way to measure the error between its own activity and the target level, and mechanisms to modulate either its neurons’ intrinsic properties or the synaptic strengths between them.

To better understand the molecular mechanisms of network regulation, we are looking at the recovery process in the pyloric network when it is treated with an enzyme that degrades a component of the neurons’ extracellular matrix called chondrotin sulfate proteoglycans (CSPGs). In vertebrates, perineuronal CSPGs are known to be found in cell-type specific, extracellular matrix aggregates whose formation is activity-dependent [2], but perineuronal CSPGs have not been studied before in invertebrates. Unpublished results show that when the enzyme is added before decentralization, the network continues to produce normal output. However, when the network is then decentralized, it does not recover like control preparations. To see if the effects of degrading the CSPGs are mediated by changes in the intrinsic or synaptic properties of the network, we turned to modeling.

Using a previously published single neuron model of the STG’s recovery process [3], we have found that reducing the activity-dependent conductance of one calcium current is enough to prevent recovery after decentralization. We looked at the calcium current because CSPGs have been shown to interact with calcium channels in other systems [4]. When the model neuron is decentralized, it stops producing its slow-wave oscillations, which leads to a decreased intracellular calcium concentration. In response, the calcium sensor upregulates the maximal calcium conductance such that bouting occurs. Full recovery occurs after another compensatory mechanism begins at a later time. As we gradually reduced the amount of calcium conductance that could be upregulated, we found that interbout periods of the bouting phase became progressively longer, until a point where bouts did not occur at all, and full recovery could not occur. We then placed a neuron with these regulatory mechanisms into a network model such as those in [5] and found that regulating the intrinsic properties of one component of the network is enough to determine whether the network produces rhythmic activity or not. Using this network model, we can also see how regulatory mechanisms that affect synaptic strengths can lead to or prevent recovery. Understanding how changes in the network’s properties leads to changes in its output will help us to narrow the search for molecular mechanisms of regulation.
